# The patterns of genomic variances and covariances across genome for milk production traits between Chinese and Nordic Holstein populations

**DOI:** 10.1186/s12863-017-0491-9

**Published:** 2017-03-15

**Authors:** Xiujin Li, Mogens Sandø Lund, Luc Janss, Chonglong Wang, Xiangdong Ding, Qin Zhang, Guosheng Su

**Affiliations:** 1 Center for Quantitative Genetics and Genomics, Department of Molecular Biology and Genetics, Aarhus University, Tjele, Denmark; 2 Laboratory of Animal Genetics, Breeding and Reproduction, Ministry of Agriculture of China, National Engineering Laboratory for Animal Breeding, College of Animal Science and Technology, China Agricultural University, Beijing, 100193 China; 3 State Key Laboratory of Biocontrol, School of Life Sciences, Guangzhou Higher Education Mega Center, Sun Yat-sen University, North Third Road, Guangzhou, Guangdong 510006 People’s Republic of China; 4 Department of Pig Genetics and Breeding, Institute of Animal Husbandry and Veterinary Medicine, Anhui Academy of Agricultural Sciences, Hefei, 230031 China

**Keywords:** Chinese Holstein, Nordic Holstein, Genomic variance, Genomic covariance, Genomic correlation

## Abstract

**Background:**

With the development of SNP chips, SNP information provides an efficient approach to further disentangle different patterns of genomic variances and covariances across the genome for traits of interest. Due to the interaction between genotype and environment as well as possible differences in genetic background, it is reasonable to treat the performances of a biological trait in different populations as different but genetic correlated traits. In the present study, we performed an investigation on the patterns of region-specific genomic variances, covariances and correlations between Chinese and Nordic Holstein populations for three milk production traits.

**Results:**

Variances and covariances between Chinese and Nordic Holstein populations were estimated for genomic regions at three different levels of genome region (all SNP as one region, each chromosome as one region and every 100 SNP as one region) using a novel multi-trait random regression model which uses latent variables to model heterogeneous variance and covariance. In the scenario of the whole genome as one region, the genomic variances, covariances and correlations obtained from the new multi-trait Bayesian method were comparable to those obtained from a multi-trait GBLUP for all the three milk production traits. In the scenario of each chromosome as one region, BTA 14 and BTA 5 accounted for very large genomic variance, covariance and correlation for milk yield and fat yield, whereas no specific chromosome showed very large genomic variance, covariance and correlation for protein yield. In the scenario of every 100 SNP as one region, most regions explained <0.50% of genomic variance and covariance for milk yield and fat yield, and explained <0.30% for protein yield, while some regions could present large variance and covariance. Although overall correlations between two populations for the three traits were positive and high, a few regions still showed weakly positive or highly negative genomic correlations for milk yield and fat yield.

**Conclusions:**

The new multi-trait Bayesian method using latent variables to model heterogeneous variance and covariance could work well for estimating the genomic variances and covariances for all genome regions simultaneously. Those estimated genomic parameters could be useful to improve the genomic prediction accuracy for Chinese and Nordic Holstein populations using a joint reference data in the future.

**Electronic supplementary material:**

The online version of this article (doi:10.1186/s12863-017-0491-9) contains supplementary material, which is available to authorized users.

## Background

The Chinese Holstein has been formed in the process that the imported Holstein bulls from Europe and North America were crossbred with local yellow cattle, and the crossbred cows were continuously back-crossed with imported Holstein bulls [1, 2]. Therefore, it is assumed that the Chinese Holstein population is genetically close to the other Holstein populations in the world. To date, Chinese and Nordic Holstein populations have been jointly studied widely [3–5]. It has been reported that around 30% of Chinese cows has a relationship coefficient above 0.20 with one or more Nordic Holstein bulls, calculated by using genomic data [5]. Zhou et al. [4] reported that the extent of linkage disequilibrium (LD) was similar in the Chinese and Nordic Holstein populations, and the consistency of LD phase between two populations was very high with a correlation of 0.97. Thus, it was successful in improving the prediction accuracy for Chinese Holstein population using a joint reference population including Nordic genotyped progeny-test bulls. In addition, the accuracy of imputation from the 54 K to the HD marker data for Chinese Holsteins was improved by adding the Nordic HD-genotyped bulls into the reference data [5].

Because of different production systems between China and Nordic countries, some genes may show significant different effects for the same trait between Chinese and Nordic Holstein populations, i.e., genotype by environment interactions. The different genetic effects on the same trait between two populations can be reflected by the patterns of genomic variances, covariances and correlations across genome, which can be detected by an analysis where a given biological trait in Chinese and Nordic Holstein populations is considered as two traits. On one hand, knowing these genomic parameters of different genome regions in two populations will give a better opportunity to understand genetic architectures of traits of interest, On the other hand, these genomic parameters can be used to improve the accuracy of genomic prediction for traits of interest in both populations when using a joint reference population.

With the availability of SNP chips and genome sequencing, SNP information has offered a possibility to study genetic architecture of complex traits. Using SNP data, it is possible to disentangle the pattern of genomic variance and covariance across the whole genome. However, there are few literatures to report genomic variances, covariances and correlations for different genome regions by using SNP information. Recently, Janss [6] has proposed a multi-trait Bayesian method using latent variables to model heterogeneous variances and covariances, which makes it easy to estimate the genomic variances and covariances for all genome regions simultaneously. Furthermore a modified model has been developed in the present study, which is more flexible to handle heterogeneous variances and covariances. So far, this new multi-trait Bayesian method has not been applied in large-scale real data.

Therefore, the objective of the present study is to investigate region-specific genomic variances in Chinese and Nordic Holstein populations as well as region-specific genomic covariances and correlations between the two populations for three milk production traits, using the novel multi-trait Bayesian method.

## Results

As shown in Table 1, the total genomic variance, total genomic covariance and overall genomic correlation estimated from MT-GBLUP were a little higher than those estimated from MT-Bayesian rrBLUP with three different scenarios, while the residual variance estimated from MT-GBLUP was lower than those estimated from MT-Bayesian rrBLUP. The largest differences between variances estimated from MT-Bayesian rrBLUP and MT-GBLUP were for MY in Chinese population, where MT-GBLUP resulted in 2.28% larger genomic variance and 1.83% smaller residual variance than MT-Bayesian rrBLUP with all SNP as one region. The total genomic variance and covariance estimated from MT-Bayesian rrBLUP with all SNP as one region were higher than the other two scenarios. Furthermore, the total genomic variance and covariance estimated from MT-Bayesian rrBLUP with each BTA as one region were higher than those from MT-Bayesian rrBLUP with every 100 SNP as one region. In all cases, the Chinese population had much larger total genomic variance than the Nordic population for all three traits due to different scale of DRP. The overall estimated genomic correlation between the Chinese and Nordic populations was positive and higher for MY than FY and PY.

**Table 1:** Total estimated genomic variance (Va), covariance (Cov), correlation (Corr) with standard error (SE) in parentheses, heritability (h^2^), and DIC for Chinese (CN) and Nordic (NO) Holstein population in different scenarios

Method	Group^a^	Trait^b^	Va_CN	Va_NO	Cov	Corr	h^2^_CN^c^	h^2^_NO^c^	DIC^d^
MT-GBLUP	All SNP	MY	358160.4 (25519.1)	112.3(4.1)	4116.0(289.4)	0.649(0.037)	0.41	0.86	NA
		FY	469.8(33.0)	104.6(3.8)	134.7(10.1)	0.607(0.038)	0.42	0.86	NA
		PY	309.0(22.3)	107.1(3.9)	98.2(8.5)	0.540(0.042)	0.40	0.86	NA
MT-rrBLUP	All SNP	MY	349985.3(27058.4)	108.2(3.8)	3825.6(271.7)	0.622(0.034)	0.40	0.84	125746.4
		FY	462.2(34.4)	102.2(3.8)	126.5(11.5)	0.582(0.044)	0.42	0.85	83081.2
		PY	302.1(21.5)	104.4(3.6)	93.6(8.5)	0.527(0.043)	0.39	0.84	81589.5
	BTA	MY	346995.3(27762.0)	104.7(5.0)	3550.9(306.1)	0.589(0.042)	0.40	0.84	125726.5
		FY	418.2(36.6)	95.2(5.1)	112.0(12.0)	0.562(0.048)	0.39	0.83	83255.6
		PY	289.5(20.8)	101.6(4.4)	90.8(8.1)	0.529(0.040)	0.38	0.83	81554.3
	100 SNPs	MY	279770.6(23729.6)	85.4(3.7)	2856.8(281.3)	0.585(0.044)	0.35	0.81	125667.4
		FY	331.4(26.9)	74.5(4.1)	82.2(7.3)	0.523(0.037)	0.34	0.80	83143.4
		PY	272.5(18.1)	86.6(4.1)	85.9(8.0)	0.559(0.044)	0.36	0.81	81570.8

^a^ *All SNP* the whole genome, *BTA,* each chromosome as one genome region, *100 SNPs* every 100 SNP as one genome region

^b^ *MY* Milk Yield, *FY,* Fat Yield, *PY* Protein Yield

^c^ *h* ^*2*^ represented the reliability of DRP

^d^ *DIC* Deviance Information Criterion

The proportion of additive genetic variance to phenotypic variance (h^2^) in Table 1 represented reliability of DRP. The reliabilities in Nordic Holsteins were much higher than those in Chinese Holsteins, because the Nordic Holsteins in the analysis comprised bulls with large group of daughters while the Chinese Holsteins comprised mainly cows. In addition, based on the Deviance Information Criterion (DIC) statistical criteria, MT-Bayesian rrBLUP with every 100 SNP as one region was better than other two scenarios for MY, MT-Bayesian rrBLUP with all SNP as one region was best among three scenarios for FY, and MT-Bayesian rrBLUP with each BTA as one region was best for PY.

**Fig. 1:**
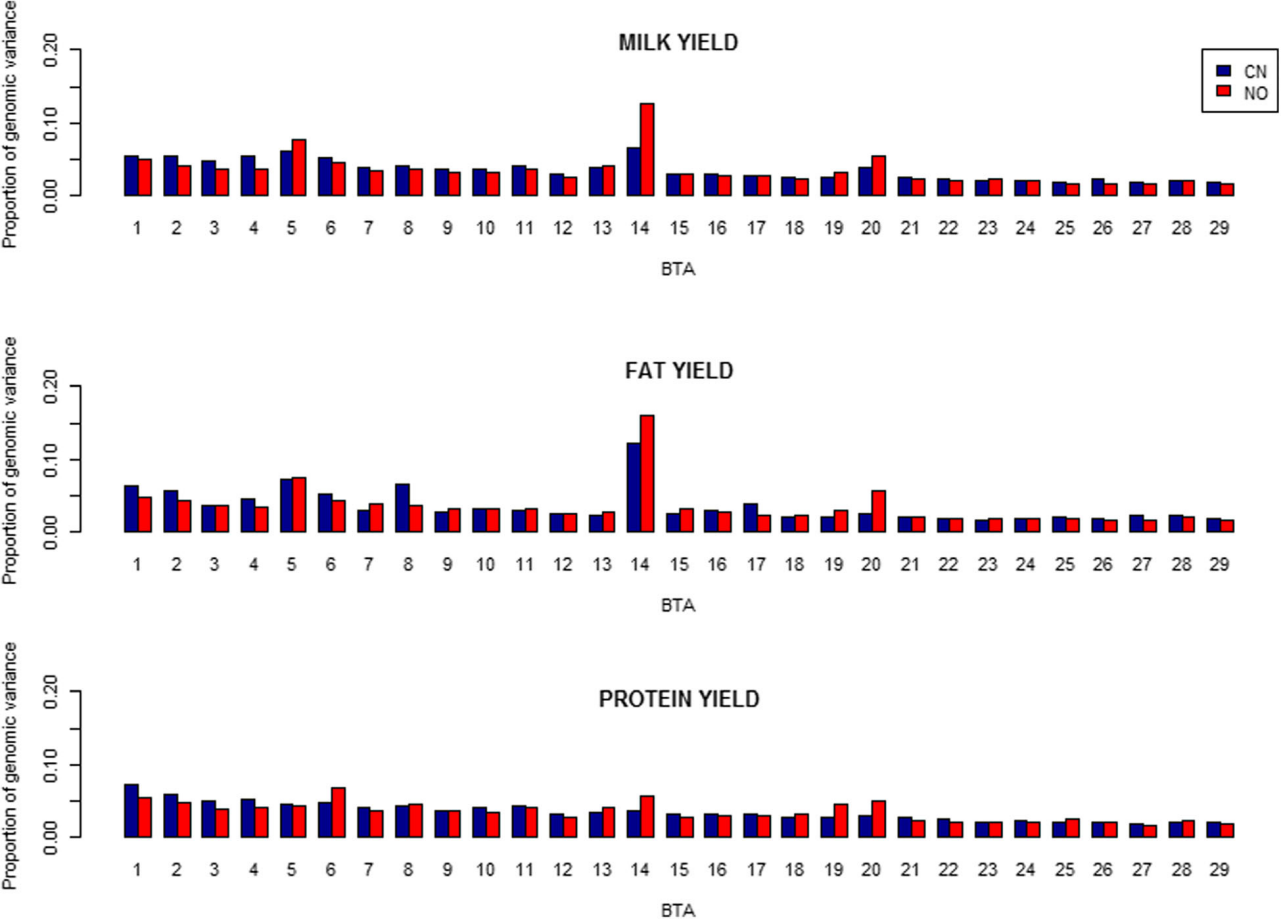
Distribution of proportions of total genomic variances explained by each chromosome for three traits in Chinese (CN) and Nordic (NO) Holstein populations in the scenario of each chromosome as one genome region

Figure 1 shows the distribution of 29 chromosome-wide genomic variances in each population. For MY and FY, BTA 14 and BTA 5 explained the highest and the second highest proportion of genomic variance in both populations. For example, BTA 14 explained 6.48% for MY and 12.73% for FY in the Chinese population, 12.69% for MY and 15.92% for FY in the Nordic population, respectively. In the Nordic population, BTA 20 also explained the third highest proportion of genomic variance (i.e., 5.33% for MY and 5.65% for FY). For PY, in the Chinese population, the proportion of genomic variance from BTA 1 to BTA 29 had a strong linear relation (i.e., *R* ^2^=0.97) with the length (in bp) of individual chromosome, while in the Nordic population, BTA 1, BTA 6, BTA 14, and BTA 20 explained larger proportions of genomic variance (i.e., 5.44, 6.76, 5.69 and 4.99%) than other BTAs.

**Fig. 2:**
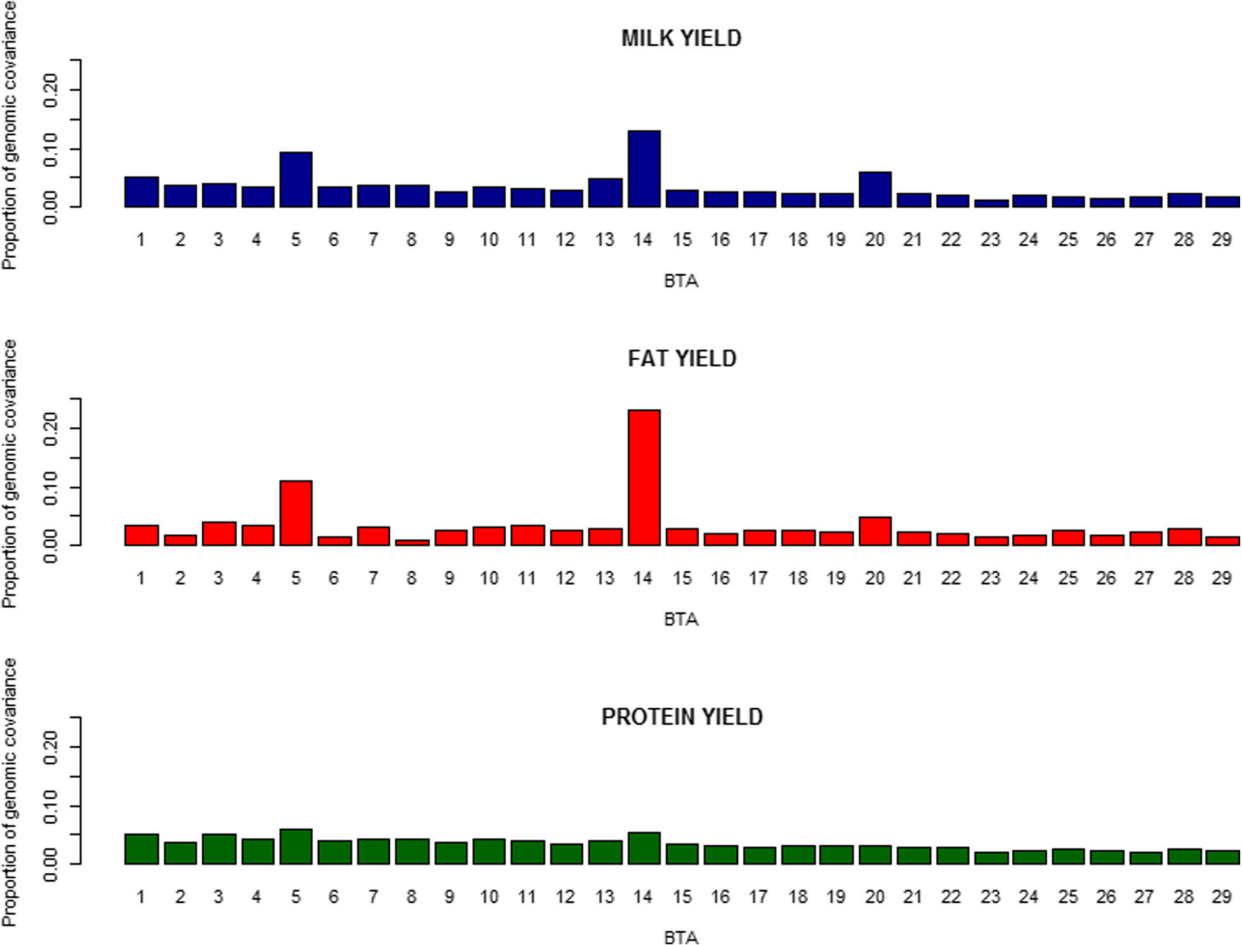
Distribution of proportions of total genomic covariances explained by each chromosome between Chinese and Nordic Holstein populationsfor three traits in the scenario of each chromosome as one genome region

**Fig. 3:**
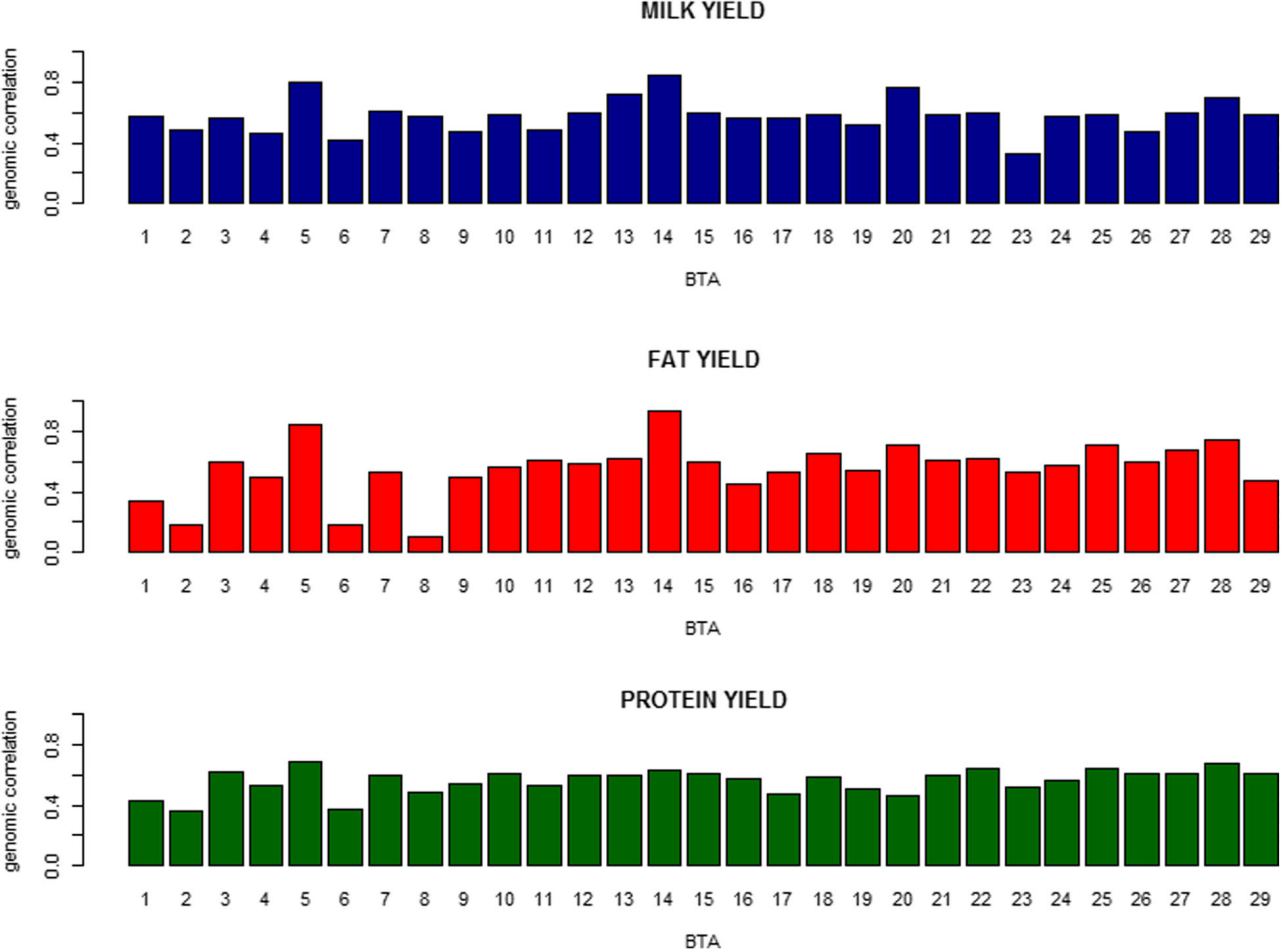
Distribution of genomic correlations for three traits between Chinese and Nordic Holstein populations in the scenario of each chromosome as one genome region

As presented in Fig. 2, for PY, the proportion of genomic covariance between the two populations ranged from 1.93% (BTA 27) to 5.79% (BTA 5), and had a linear relation (*R* ^2^ = 0.80) with the length of individual chromosome. However, for both MY and FY, similar to the pattern of genomic variance, BTA 14, BTA 5 and BTA 20 explained much larger proportions (i.e., 13.02, 9.26 and 5.87% for MY and 23.16, 11.07 and 4.66% for FY) than other chromosomes. As seen in Fig. 3, genomic correlations ranged from 0.320 (BTA 23) to 0.845 (BTA 14) for MY, from 0.096 with (BTA 8) to 0.937(BTA 14) for FY, and from 0.358 (BTA 2) to 0.690 (BTA 5) for PY. Similar to the patterns of genomic variance and covariance, BTA 14 and BTA 5 also showed much higher genomic correlations for MY and FY than other chromosomes, i.e., 0.845 and 0.801 for MY and 0.937 and 0.851 for FY, respectively.

**Fig. 4:**
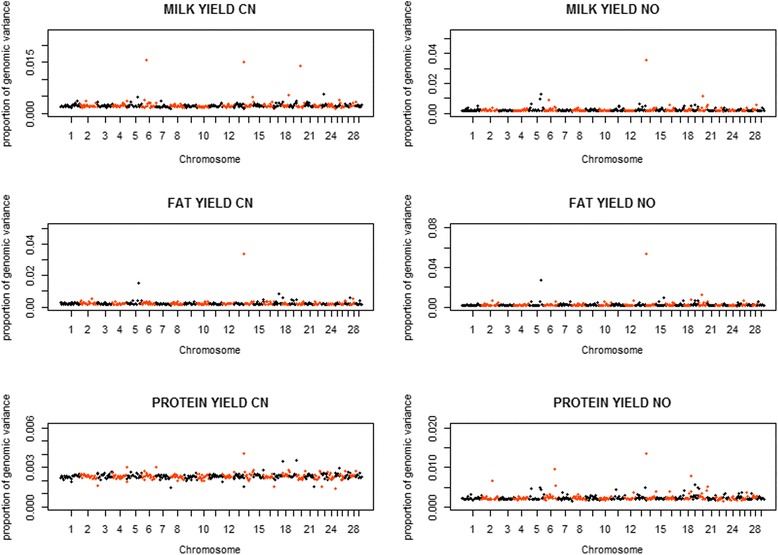
Distribution of proportions of genomic variances explained by chromosome regions of 100 SNP for three traits in Chinese (CN) and Nordic (NO) Holstein populations

Besides the scenario of each chromosome as one genome region, we also divided the whole genome into 430 regions with every adjacent 100 SNP as one genome region. The distribution of proportions of genomic variances explained by each region of 100 SNP for three traits is shown in Fig. 4. In the Chinese Holstein population, most regions explained <0.50% of the total genomic variance for MY and FY. There were also 5 regions explaining 0.53 to 1.57% for MY and 7 regions explaining 0.53 to 3.38% for FY. For PY, most regions explained <0.30% of the total genomic variance, and there were 5 regions explaining larger proportions of variance, ranging from 0.30 to 0.41%. Similar to the Chinese population, in the Nordic population, most regions explained < 0.50% of the total genomic variance for MY and FY, and explained < 0.30% for PY. However, there were 14 regions explaining 0.50 to 3.57% for MY, 19 regions explaining 0.51 to 5.38% for FY, and 35 regions explaining 0.30 to 1.36% for PY.

**Fig. 5:**
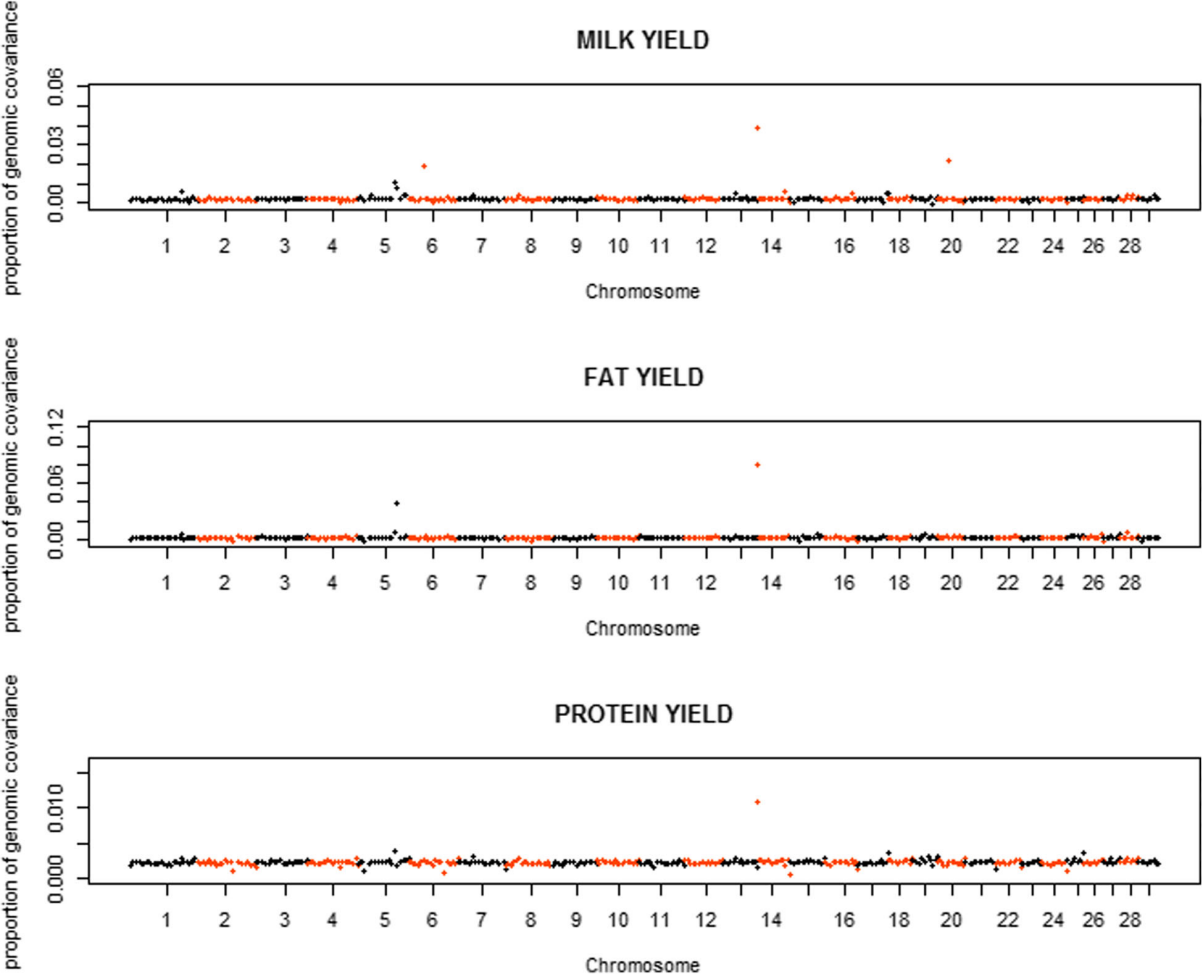
Distribution of proportions of genomic covariances explained by chromosome regions of 100 SNP between Chinese and Nordic Holstein populationsfor three traits

**Fig. 6:**
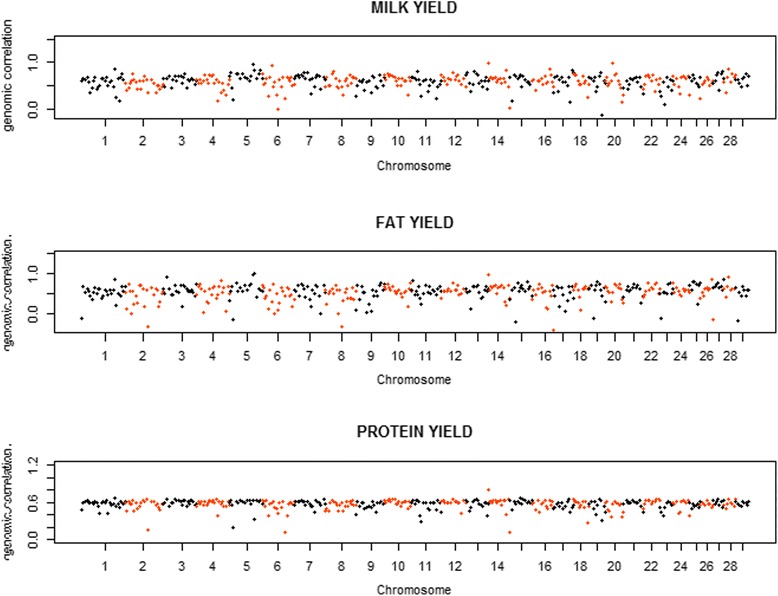
Distribution of genomic correlations explained by chromosome regions of 100 SNP between Chinese and Nordic Holstein populationsfor three traits

Figure 5 presents the distribution of the proportions of genomic covariances between the two populations explained by each region of 100 SNP for all three traits. Most regions explained < 0.50% of the total genomic covariance for MY and FY, and explained < 0.30% of the total genomic covariance for PY. Meanwhile, there were also 10 regions explaining 0.50 to 3.90% for MY, 9 regions explaining 0.50 to 8.03% for FY, and 9 regions explaining 0.30 to 1.08% for PY, respectively. As seen in Fig. 6, the estimates of genetic correlations ranged from −0.137 to 0.992 for MY, from −0.411 to 0.994 for FY and from 0.111 to 0.811 for PY. Most of the genomic correlations ranged from 0.40 to 0.70, and there were 1 region for MY and 10 regions for FY showing highly negative genomic correlations.

## Discussion

The present study for the first time reported the patterns of genomic variance, covariance and correlation between the Chinese and Nordic Holstein populations for three milk production traits. The patterns of those genomic parameters were investigated at different levels of genome regions, i.e., the whole genome as one genome region, each chromosome as one region and every 100 SNP as one region, using the novel MT-Bayesian rrBLUP model. The results showed that the MT-Bayesian rrBLUP worked well for estimating genomic parameters for different regions in the genome simultaneously. As expected, the results showed that different regions explained different amounts of genomic variance and covariance as well as different degrees of genomic correlation.

Both MT-GBLUP and MT-Bayesian rrBLUP were used to estimate genomic variance, genomic covariance and residual variance between Chinese and Nordic Holstein populations for three milk production traits. The total genomic variance and covariance as well as genomic correlation estimated from MT-GBLUP were slightly higher than those from MT-Bayesian rrBLUP without dividing the genome into regions. Although MT-GBLUP and MT-rrBLUP without dividing regions were equivalent, the two models used different algorithms. The MT-GBLUP used AI-REML algorithm while MT-rrBLUP used MCMC algorithm. The two different algorithms might lead to slight difference in estimates of variance and covariances, especially when the distribution of the estimates was deviated from a normal distribution. In fact, the difference was very small, compared with the size of the standard errors. MT-Bayesian rrBLUP had the advantage to estimate region-specific variance and covariance for all regions simultaneously. In principle, MT-GBLUP also can estimate region-specific variance and covariance for all regions simultaneously by taking each region as one component in the model, but it has a very high computational demand, and is difficult to reach convergence due to too many components in the model. Another approach to use MT-GBLUP to estimate region-specific variance and covariance is to take a particular region as one component and the others as one component in the model, and thus obtain variance and covariance for one region in the model in one run. But this is still a time-consuming approach. For example for MY, although the computation time for MT-GBLUP is much shorter than MT-rrBLUP (3 h vs 42 h), the time for MT-GBLUP with every 100 SNP as one region is much longer than MT-rrBLUP with every 100 SNP as one region (10 h* region number vs 42 h). Thus it is difficult to use MT-GBLUP to estimate regional specific variance and covariance with every 100 SNP as one region because of very time consuming. It was observed that with the number of genome regions increased, total genomic variance and covariance derived from each region decreased (Table 1). One possible reason could be that total genomic variance and covariance were obtained by simply summing the genomic variance and covariance for each region, and ignoring covariance between regions. In addition, the correlations of genomic estimated breeding values (GEBV) between MT-Bayesian rrBLUP with three different scenarios ranged from 0.87 to 0.94 for MY, from 0.80 to 0.91 for FY, and from 0.96 to 0.98 for PY in both populations. These showed that the performance of different MT-Bayesian rrBLUP with different assumptions is greatly influenced by genetic architectures of traits of interest.

At chromosome level, very large genomic variance, covariance and correlation for three traits were found on BTA14 as the *diacylg-lycerolacyltransferase 1* (*DGAT1*) on BTA 14 has a very large effect and segregates in the Holstein populations [7–9]. Among the regions with size of 100 SNP on BTA 14, the genome region (1.46 Mbp ~ 5.57 Mbp) showed much higher genomic variance, covariance and correlation (0.98 for MY and FY; 0.80 for PY) than other regions, which confirmed that *DGAT1* plays an important role on these three traits (Additional file 1: Figure S1 and Additional file 2: Figure S2) in both Chinese and Nordic Holstein populations. On the other hand, the proportions of genomic variance and covariance for this first region for PY were smaller than those for the other two traits, which indicated that the effect of *DGAT1* was larger on MY and FY than on PY.

Besides BTA 14, BTA 5 and BTA 20 also showed a relative high genomic covariance and correlation which speculated that some QTL regions had the same effects one those two chromosomes in both populations. Among all 100 SNP regions on BTA 5, two consecutive regions (84.09 Mbp ~ 98.29 Mbp, 13^th^ and 14^th^region on BTA5) showed much higher genomic variance, covariance and correlation than other regions for MY and FY (Additional file 3: Figure S3 and Additional file 4: Figure S4). This segment on BTA 5 has been previously reported to include the QTL affecting MY and FY in Holstein populations [7, 10, 11]. In the present study, we also detected that aregion (27.93 Mbp ~ 34.73 Mbp), which included the *growth hormone receptor* (*GHR*) gene, presented much higher genomic variance, genomic covariance and genomic correlation for MY in this region than other regions on BTA 20 (Additional file 5: Figure S5). It has been reported that the *growth hormone receptor* (*GHR*) gene on BTA 20 has a large effect on MY in Holstein populations [8, 12–14].

Although overall genomic correlations between two populations for three traits were highly positive, some genome regions showed weakly positive correlation or highly negative genomic correlations for MY and FY. In the present study, we found one region (47.20 Mbp ~52.23 Mbp) on BTA 19 for MY and 10 regions on different chromosomes for FY showing highly negative correlations (Additional file 6: Table S1). There were two regions (48.79 Mbp–53.49 Mbp; 58.99 Mbp–64.03 Mbp) on BTA 8 showing lower genomic covariance and negative genomic correlation, i.e., −0.016 and −0.327 (Additional file 7: Figure S6), and thus these two region might resulted in the lowest genomic covariance and correlation for BTA8 among all autosomes for FY (Figs. 2 and 3). The negative genomic correlations between two populations indicated that the same region has different effects on the same trait in different populations. One possible reason for some regions showing negative correlations could be due to different selection histories, e.g. different weights for traits in the selection index, between two populations which led to difference in effects between two populations for some genomic regions. Another possible reason could be due to different production systems in Chinese and Nordic Holstein populations, i.e., genotype by environment interaction. In other words, the regions with negative or very low correlations can be considered as candidate regions involved in genotype be environment interaction for further studies.

How to define genome regions to inspect the patterns of genomic variance and covariance is a key point, and it needs to be further investigated. In this study, we divided the whole genome into regions in an arbitrary way (i.e., each chromosome or every 100SNP as one region). The way to partition the genomic variance into chromosomes has also been reported by Jensen et al. [15]. For the way to define how many SNP as one region, we have tried to use every 50 SNP, every 100 SNP, every 200 SNP and every 400 SNP as one region to estimate genomic variance and covariance for each region, respectively. The results showed that every 100, 200,400 SNP as one region performed well, while every 50 SNP as one region performed worse, especially for MY, with regard to total genomic variance and covariance (results not shown). The reason may be that small regions, such as every 50 SNP as one region, reduce the accuracy of estimated variance and covariances because of less accuracy of ${\mathrm{r}}_{\mathrm{ij}}$ for a particular region j. We chose every 100 SNP as one genome region rather than every 200 or 400 SNP to analyze the region patterns of genomic variance, covariance and correlation because the large region dilutes differences in variance and covariance between regions.

Using a weighted G matrix in a single-trait GBLUP model to improve the accuracy of genomic prediction has already been reported, in which the estimated SNP variances are used as a weighting factor for building a trait-specific G matrix [16, 17]. Likewise, the information of genomic variance, covariance and correlation in our study can also be further used as weighting factors for building a weighted G matrix for MT-GBLUP so that a MT-GBLUP can account for heterogeneous variances and covariances across the genome. In the Chinese cattle population, reference data mainly comprises females. Ding et al. [18] have reported prediction accuracy using such a reference data are greatly increased, compared with using pedigree information. The genomic prediction accuracy for Chinese Holsteins has been improved by using Chinese and Nordic Holsteins as reference animals and MT-GBLUP [4, 5]. Similar to the single trait model, MT-Bayesian methods with different SNP/regions having different variances may produce higher reliability of genomic prediction than MT-GBLUP assuming that all SNP have equal variances across genome. Therefore, we will consider MT-Bayesian methods with different SNP/regions and MT-GBLUP with such a weighted G matrix to further improve the prediction accuracy for the Chinese Holstein population using a joint reference population including reference animals from other countries.

## Conclusions

This study revealed the patterns of genomic variance, covariance and correlation for genomic regions across the whole genome between Chinese and Nordic Holstein populations for three milk production traits. BTA14 and BTA 5 showed very large genomic variance, covariance and correlation for MY and FY than other chromosomes, whereas no specific chromosomes showed very large genomic variance, covariance and correlation for PY. In scenario of every 100 SNP as one genome region, most regions explained <0.50% of genomic variance and covariance for MY and FY, and most regions explained <0.30% for PY. A few regions showed highly negative genomic correlations, e.g., one important region on BTA8 for FY. These regions can be considered as candidate regions accounting for interaction between genotype and production environment for MY and FY. The pattern of genomic variance, covariance and correlation across genomic regions could be useful for improving multi-population (or multi-breed) genomic prediction.

## Methods

### Data

In this study, both Chinese and Nordic Holstein cattle were genotyped with the Illumina BovineSNP50 BeadChip (Illumina, San Diego, CA). For each population, we firstly removed SNP with unknown positions and SNP on Bostaurus (BTA) X, and then imputed missing genotypes using the software Beagle [19]. After the imputation, SNP with minor allele frequencies less than 0.01 were removed. Finally, the marker set included 43,008 SNP on 29 autosomes in both populations. In order to investigate the patterns of genomic variance, covariance and correlation across the whole genome, we divided the whole genome into genome regions in two ways, which has been done by Jensen et al. [15] and Hayes et al. [20]. The first way was that each chromosome was considered as one genome region. The second way was that every adjacent 100 SNP were considered as one region. If the last region of one BTA had less than 50 SNP, this region was merged with the previous region; otherwise, this region was considered as one independent region.

The Chinese Holstein population consisted of 237 genotyped progeny-test bulls and 6076 genotyped cows, and the Nordic population included 5244 genotyped progeny-test bulls. Three milk production traits, i.e., milk yield (MY), fat yield (FY) and protein yield (PY), were analyzed. In the analysis, deregressed proofs (DRP) were used as phenotypes for all three traits. The DRP of Chinese Holstein bulls and cows were derived from the estimated breeding values (EBV) in original scale obtained from Dairy Association of China, and the DRP of Nordic Holstein bulls were derived from the EBV in standardized scale (http://www.landbrugsinfo.dk/Kvaeg/Avl/Sider/principles.pdf) obtained from Nordic Genetic Evaluation. Thus, the DRP had different scales in two populations.

### Statistical Model

In this study, each given biological trait was regarded as two different but genetic correlated traits in the Chinese and Nordic Holstein populations. A new multi-trait Bayesian method according to Janss [6] and the modified form of this method were used to calculate overall variance and covariance as well as genomic variances, covariances and correlations for genome regions for each milk production trait in both populations, respectively. The overall variance and covariance were also estimated using a multi-trait genomic BLUP (MT-GBLUP).

### Multi-trait Bayesian rrBLUP model for homogeneous variance and covariance

Similar to the single trait ridge regression BLUP (rrBLUP) model which assumes constant variance for all SNP, the logical assumption in the multi-trait rrBLUP (MT-rrBLUP) is to assume constant variance and covariance for all SNP. The MT-Bayesian rrBLUP model (it was referred to the new multi-trait Bayesian method above because it performed using the Bayesian Markov chain Monte Carlo(MCMC)) was described as follows [6]:

1 $$ {\boldsymbol{y}}_i={u}_i+\boldsymbol{W}{\boldsymbol{a}}_i+{\boldsymbol{e}}_i, $$

where ***y***_*i*_ was a vector of phenotypic values (DRP) for the population i (i = 1,2); *u*_*i*_ was the overall mean; ***e***_*i*_ was the random residual and was uncorrelated between two populations. It was assumed that ${\boldsymbol{e}}_i\sim \mathrm{N}\left(0,{\boldsymbol{D}}_i{\sigma}_{e_i}^2\right)$ in which **D**_*i*_ was a diagonal matrix with weights of the residual variance [21]; **W** was a matrix with SNP genotype covariates. The elements in **W** were (0, 1, 2)-2p_k_ for genotype A_1_A_1_, A_1_A_2_ and A_2_A_2_, where p_k_ is the frequency of minor allele A_2_ at SNP k; ***a***_*i*_ was a vector of SNP effects for the population i. SNP effects across populations were correlated by using the following hierarchical models for SNP effects:

2 $$ {\boldsymbol{a}}_i={r}_i\ast \boldsymbol{s}+{{\boldsymbol{a}}_i}^{*}, $$

The prior distributions were assumed as ${r}_i\sim u n i,\mathbf{s}\sim \mathrm{N}\left(0,\mathbf{I}\right),{{\boldsymbol{a}}_i}^{*}\sim \mathrm{N}\left(0,\mathbf{I}{\sigma_{a{ i}^{*}}}^2\right),{\sigma_{a{ i}^{*}}}^2\sim u n i,$ where the part *r*_*i*_ * ***s*** contributed to the covariance between SNP effects across populations; the “residual SNP effect” ***α***_i_^*^ was taken as uncorrelated across populations. The modeled variance and covariance for each SNP were worked out as $\operatorname{var}\left({\boldsymbol{a}}_i\right)=\operatorname{var}\left({r}_i\ast \boldsymbol{s}+{{\boldsymbol{a}}_i}^{*}\right)={r}_i^2+{\sigma_{a{ i}^{\ast}}}^2$, and cov(***a***_1_, ***a***_2_) = cov(*r*_*i*_ ∗ ***s*** + ***a***_1_^  ∗^, *r*_2_ ∗ ***s*** + ***a***_2_^  ∗^) = *r*_1_ ∗ *r*_2_. Thus, the total genomic variance for the population i was ${V}_{g_i}={\displaystyle {\sum}_{k=1}^{nSNP}2{p}_{i k}\ast \left(1-{p}_{i k}\right)* var\left({\boldsymbol{a}}_i\right)}$, the total covariance between two populations was ${V}_{g_{1,2}}={\displaystyle {\sum}_{k=1}^{nSNP}2*\sqrt{p_{1 k}*\left(1-{p}_{1 k}\right)*{p}_{2 k}*\left(1-{p}_{2 k}\right)}* cov\left({\boldsymbol{a}}_1,{\boldsymbol{a}}_2\right)}$, and the overall genomic correlation was ${R}_{g_1{g}_2}=\frac{V_{g_{1,2}}}{\sqrt{V_{g_1}*{V}_{g_2}}},$ where nSNP was the total number of SNP across the genome; ${\mathrm{p}}_{\mathrm{ik}}$ was the frequency of minor allele at marker locus k in the population i.

### MT-Bayesian rrBLUP model for heterogeneous variances and covariances

In order to calculate genomic variances, covariances and correlations for different genome regions, multi-trait Bayesian model proposed by Janss [6] was extended. The assumption for the new MT-Bayesian rrBLUP model was that effects of all SNP in each genome region have the same genomic variance and covariance, but effects of SNP in different regions have different genomic variances and covariances. The model was described as follows:

3 $$ {\boldsymbol{y}}_i={u}_i+{\displaystyle {\sum}_{j=1}^{ngroup}{\boldsymbol{W}}_j{\boldsymbol{a}}_{i j}+{\boldsymbol{e}}_i,} $$

where **y**_i_, u_i_, and **e**_i_ were the same as model 1; ngroup was the total number of genome regions; **W**_j_ was a matrix with SNP genotype covariates for the region j; **a**_ij_ was a vector of SNP effects for the region j in the population i. For the region j, SNP effects across populations were correlated and were formulated by the following hierarchical models:

4 $$ {\boldsymbol{a}}_{i j}={r}_i*{\boldsymbol{s}}_0+{r}_{i j}*{\boldsymbol{s}}_1+{{\boldsymbol{a}}_{i j}}^{*}, $$

The prior distributions were assumed as

$$ \begin{array}{c}{r}_i\sim uni,{\boldsymbol{s}}_0\sim \mathrm{N}\left(0,\mathbf{I}\right),\\ {}{r}_i\sim \mathrm{N}\left(0,{\sigma_{r_i}}^2\right),{\sigma_{r_i}}^2\sim uni,{\boldsymbol{s}}_1\sim \mathrm{N}\left(0,\mathbf{I}\right)\\ {}{{\boldsymbol{a}}_{i j}}^{*}\sim \mathrm{N}\left(0,\mathbf{I}{\sigma_{{a_i}^{*}}}^2\right),{\sigma_{r_i}}^2\sim uni.\end{array} $$

Thus, the genomic variance for each SNP in region j in population i (i = 1, 2) was worked out as $\operatorname{var}\left({\boldsymbol{a}}_{i j}\right)={r}_i^2+{r}_{i j}^2+{\sigma_{a{ i}^{*}}}^2,$ and the genomic covariance between the two populations was cov(***a***_1*j*_, ***a***_2*j*_) = *r*_1_ * *r*_2_ + *r*_1*j*_ * *r*_2*j*_. Therefore, the total genomic variance for the region j in the population i was ${V}_{g_{ij}}={\displaystyle {\sum}_{k=1}^{nSNP}2{p}_{ik}\ast \left(1-{p}_{ik}\right)*\operatorname{var}\left({\boldsymbol{a}}_{ij}\right),}$ the total genomic covariance for the region j between two populations was ${V}_{g1 j,2 j}={\displaystyle {\sum}_{k=1}^{nSNP}2*\sqrt{p_{1 k}\ast \left(1-{p}_{1 k}\right)*{p}_{2 k}*\left(1-{p}_{2 k}\right)}* cov\left({\boldsymbol{a}}_{1 j},{\boldsymbol{a}}_{2 j}\right),}$ and the overall genomic correlation for the region j was ${R}_{g1 j,2 j}=\frac{ V g1 j,2 j}{\sqrt{V{g}_{1 j}* V{g}_{2 j}}},$ where nSNP was the total number of SNP for region j; *p*_*ik*_ was the frequency of minor allele at marker locus k in the population i. The proportion of genomic variance and covariance explained by each region were calculated as ${V}_{g_{ij}}/{\displaystyle {\sum}_{j=1}^{ngroup}{V}_{g_{ij}}}$ and *V*_*g*1*j*,2*j*_/∑_*j* = 1_^*ngroup*^ *V*_*g*1*j*,2*j*_, respectively.

The analysis of MT-Bayesian rrBLUP model was carried out using the BAYZ software (www.bayz.biz). The Markov chains were run for 50,000 cycles of Gibbs sampling, and the first 20,000 cycles were discarded as burning in. After this, every 20th sample of the remaining 30,000 cycles was saved for the posterior analysis, and then median value was considered as the estimate of each unknown parameter. The posterior standard deviations of estimated genetic parameters across 30,000 cycles were denoted as standard error.

### Multi-trait GBLUP model

A multi-trait linear model (MT-GBLUP) was used to compare with the MT-Bayesian rrBLUP model with regard to overall variance and covariance. The MT-GBLUP analysis was carried out using average information restricted maximum likelihood (AI-REML) algorithm implemented in the DMU package [22]. The model was described as follows:

5 $$ \left[{}_{{\boldsymbol{y}}_{\mathbf{2}}}^{{\boldsymbol{y}}_{\mathbf{1}}}\right]=\left[{}_{u_2}^{u_1}\right]+\left[{{}_{\mathbf{0}}^{{\boldsymbol{Z}}_{\mathbf{1}}}}_{{\boldsymbol{Z}}_{\mathbf{2}}}^{\mathbf{0}}\right]\left[{}_{{\mathbf{a}}_{\mathbf{2}}}^{{\mathbf{a}}_{\mathbf{1}}}\right]+\left[{}_{{\boldsymbol{e}}_{\mathbf{2}}}^{{\boldsymbol{e}}_{\mathbf{1}}}\right] $$

where ***y***_**1**_ and ***y***_**2**_ were DRP of Chinese and Nordic populations, respectively; *u*_1_ and *u*_2_ were overall mean of DRP of Chinese and Nordic populations; $\left[{}_{{\mathbf{a}}_{\mathbf{2}}}^{{\mathbf{a}}_{\mathbf{1}}}\right]$ was a vector of additive genetic effects; ***Z***_**1**_ and ***Z***_**2**_ were incidence matrices linking **a**_**1**_ to ***y***_**1**_ and **a**_**2**_ to ***y***_**2**_, respectively; and ***e***_**1**_ and ***e***_**2**_ were vectors of residuals for ***y***_1_ and ***y***_2_ respectively. It was assumed that $\left[{}_{{\mathbf{a}}_{\mathbf{2}}}^{{\mathbf{a}}_{\mathbf{1}}}\right]\sim \mathrm{N}\left(0,{\boldsymbol{G}}_{\mathbf{0}}\otimes \mathbf{G}\right)$ where ***G***_0_ was a covariance matrix, i.e., $$ {\boldsymbol{G}}_{\mathbf{0}}=\left[\begin{array}{cc}\hfill {\sigma}_{a_1}^2\hfill & \hfill {\sigma}_{a_1{a}_2}\hfill \\ {}\hfill {\sigma}_{a_1{a}_2}\hfill & \hfill {\sigma}_{a2}^2\hfill \end{array}\right] $$ and the **G** matrix was constructed according to the method 1 described by VanRaden [23]; ***e***_1_ and ***e***_2_ were assumed to be uncorrelated, thus ${\boldsymbol{e}}_{\mathbf{1}}\sim \mathrm{N}\left(0,{\mathbf{D}}_{\mathbf{1}}{\sigma}_{e_1}^2\right)$ and ${e}_2\sim \mathrm{N}\left(0,{\mathbf{D}}_{\mathbf{2}}{\sigma}_{e_2}^2\right)$ in which **D**_**1**_ and **D**_**2**_ were diagonal matrices with weights for the residual variance [21].

## Additional files

Additional file 1: Figure S1. Distribution of proportions of genomic variances explained by chromosome regions of 100 SNP for three traits on BTA 14 in Chinese (CN) and Nordic (NO) Holstein populations. (TIFF 1122 kb)
Additional file 2: Figure S2. Distribution of proportions of genomic covariances and genomic correlations explained by chromosome regions of 100 SNP for three traits on BTA 14 between Chinese and Nordic Holstein populations. (TIFF 1122 kb)
Additional file 3: Figure S3. Distribution of proportions of genomic variances explained by chromosome regions of 100 SNP for three traits on BTA 5 in Chinese (CN) and Nordic (NO) Holstein populations. (TIFF 1122 kb)
Additional file 4: Figure S4. Distribution of proportions of genomic covariances and genomic correlations explained by chromosome regions of 100 SNP for three traits on BTA 5 between Chinese and Nordic Holstein populations. (TIFF 1122 kb)
Additional file 5: Figure S5. Distribution of proportions of genomic variances, covariances and genomic correlations explained by chromosome regions of 100 SNP for milk yield on BTA 20in Chinese (CN) and Nordic (NO) Holstein populations. (TIFF 1122 kb)
Additional file 6: Table S1. The regions of 100SNP showing highly negative genomic correlations for milk yield and fat yield. (XLSX 10 kb)
Additional file 7: Figure S6. Distribution of proportions of genomic variances, covariances and genomic correlations explained by chromosome regions of 100 SNP for fat yield on BTA 8 in Chinese (CN) and Nordic (NO) Holstein populations. (TIFF 1122 kb)

## Abbreviations

DRP: Deregressed proofs
EBV: Estimated breeding values
FY: Fat yield
MT-Bayesian rrBLUP: Multi-trait Bayesian ridge regression BLUP
MT-GBLUP: Multi-trait genomic BLUP
MY: Milk yield
PY: Protein yield
