# Supplementary figures and images for: The patterns of genomic variances and covariances across genome for milk production traits between Chinese and Nordic Holstein populations
Source: BMC Genet. 2017 Mar 15;18:26. doi: 10.1186/s12863-017-0491-9 (PMC5353867; doi:10.1186/s12863-017-0491-9)

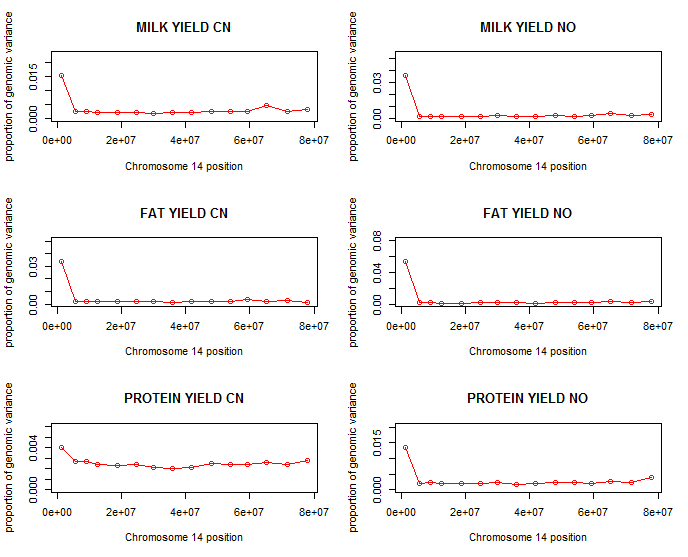

Supplement: Additional file 1: Figure S1. — Distribution of proportions of genomic variances explained by chromosome regions of 100 SNP for three traits on BTA 14 in Chinese (CN) and Nordic (NO) Holstein populations. (TIFF 1122 kb) [file 12863_2017_491_MOESM1_ESM.tiff]

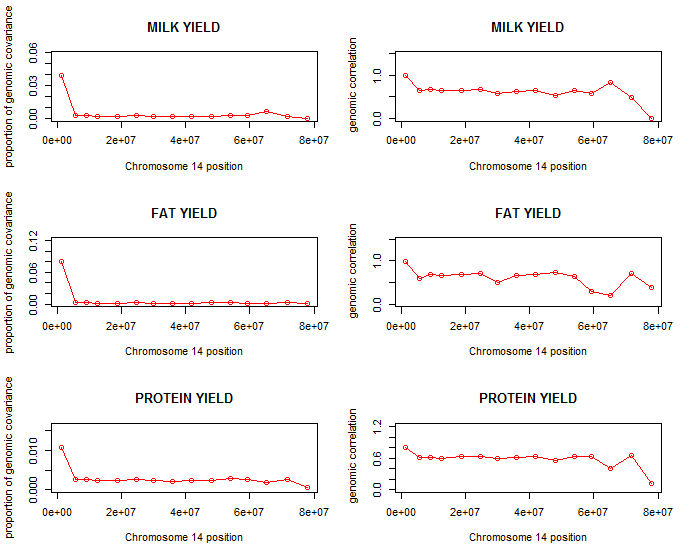

Supplement: Additional file 2: Figure S2. — Distribution of proportions of genomic covariances and genomic correlations explained by chromosome regions of 100 SNP for three traits on BTA 14 between Chinese and Nordic Holstein populations. (TIFF 1122 kb) [file 12863_2017_491_MOESM2_ESM.tiff]

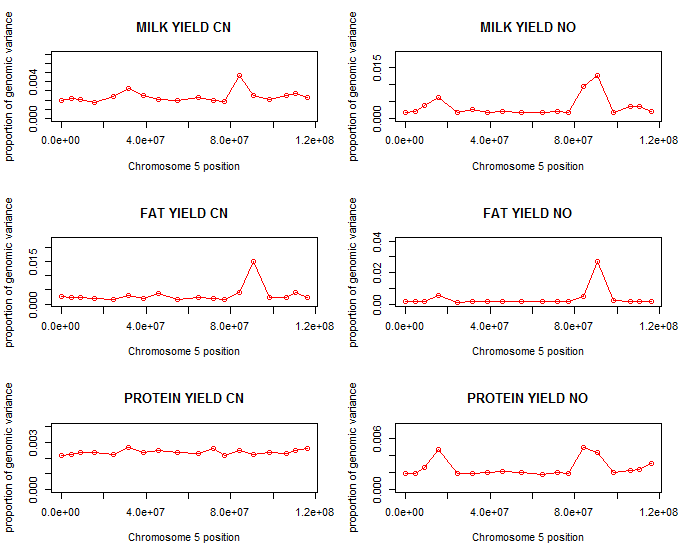

Supplement: Additional file 3: Figure S3. — Distribution of proportions of genomic variances explained by chromosome regions of 100 SNP for three traits on BTA 5 in Chinese (CN) and Nordic (NO) Holstein populations. (TIFF 1122 kb) [file 12863_2017_491_MOESM3_ESM.tiff]

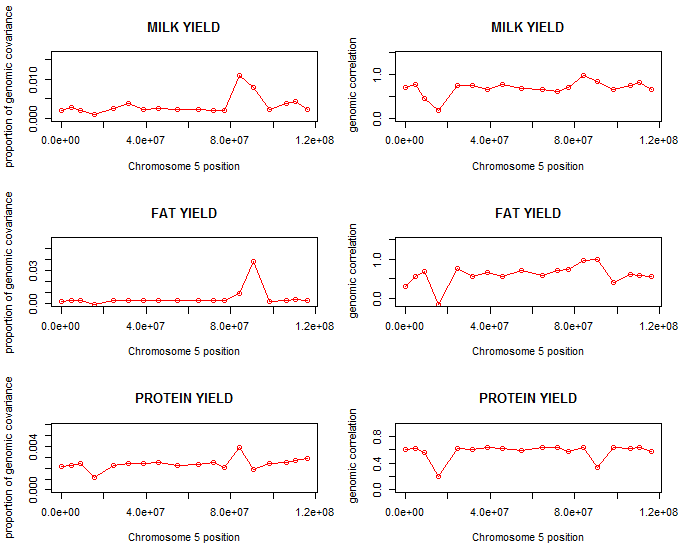

Supplement: Additional file 4: Figure S4. — Distribution of proportions of genomic covariances and genomic correlations explained by chromosome regions of 100 SNP for three traits on BTA 5 between Chinese and Nordic Holstein populations. (TIFF 1122 kb) [file 12863_2017_491_MOESM4_ESM.tiff]

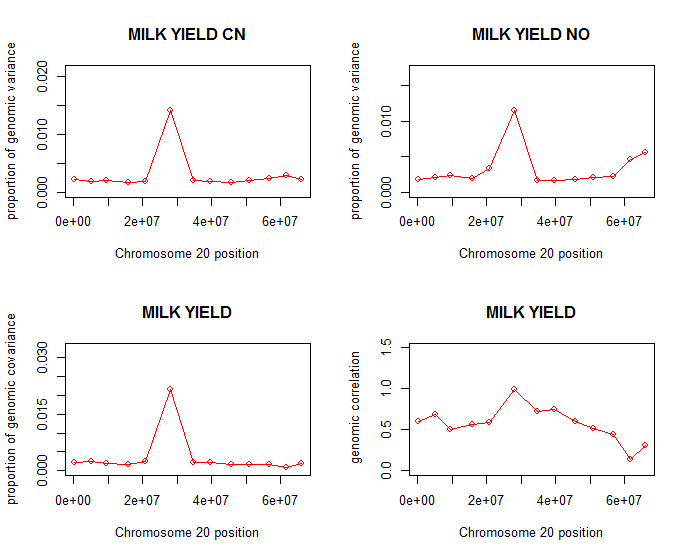

Supplement: Additional file 5: Figure S5. — Distribution of proportions of genomic variances, covariances and genomic correlations explained by chromosome regions of 100 SNP for milk yield on BTA 20in Chinese (CN) and Nordic (NO) Holstein populations. (TIFF 1122 kb) [file 12863_2017_491_MOESM5_ESM.tiff]

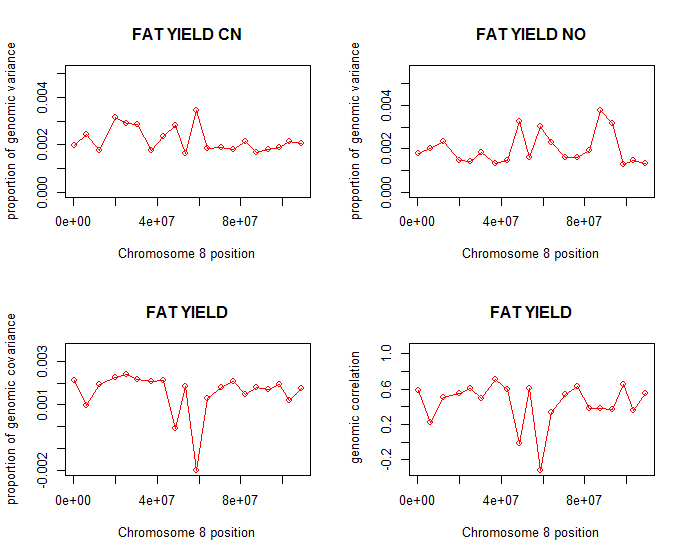

Supplement: Additional file 7: Figure S6. — Distribution of proportions of genomic variances, covariances and genomic correlations explained by chromosome regions of 100 SNP for fat yield on BTA 8 in Chinese (CN) and Nordic (NO) Holstein populations. (TIFF 1122 kb) [file 12863_2017_491_MOESM7_ESM.tiff]
